# Atherosclerotic Cardiovascular Disease in Gulf War Veterans in Relation to Deployment Exposures

**DOI:** 10.1007/s12012-025-10013-7

**Published:** 2025-07-05

**Authors:** Sarah T. Ahmed, Ruosha Li, Lea Steele, Peter Richardson, Kellie Sims, Rachel Quaden, Kelly M. Harrington, Vijay Nambi, John M. Gaziano, Robert Morgan, George L. Delclos, Drew A. Helmer

**Affiliations:** 1 Center Health Services Research & Development Center for Innovations in Quality, Effectiveness, and Safety, Michael E. DeBakey VA Medical Center, Houston, TX USA; 2 Section of Health Services Research, Department of Medicine, Baylor College of Medicine, Houston, TX USA; 3 Department of Biostatistics and Data Sciences, School of Public Health, The University of Texas Health Science Center at Houston, Houston, TX USA; 4 Yudofsky Division of Neuropsychiatry, Department of Psychiatry and Behavioral Sciences, Baylor College of Medicine, Houston, TX USA; 5 Cooperative Studies Program Epidemiology Center-Durham, Durham VA Medical Center, Durham VA Health Care System, Durham, NC USA; 6 Million Veteran Program (MVP) Coordinating Center, VA Boston Healthcare System, Boston, MA USA; 7 Department of Psychiatry, Boston University Chobanian & Avedisian School of Medicine, Boston, MA USA; 8 Section of Cardiology and Cardiovascular Research, Department of Medicine, Baylor College of Medicine, Houston, TX USA; 9 Section of Cardiology, Department of Medicine, Michael E. DeBakey VA Medical Center, Houston, TX USA; 10 Department of Medicine, Harvard Medical School, Boston, MA USA; 11 Division of Aging, Department of Medicine, Brigham and Women’s Hospital, Boston, MA USA; 12 Department of Management, Policy and Community Health, School of Public Health, The University of Texas Health Science Center at Houston, Houston, TX USA; 13 Southwest Center for Occupational and Environmental Health, Department of Environmental and Occupational Health Sciences, School of Public Health, The University of Texas Health Science Center at Houston, Houston, TX USA

**Keywords:** Gulf war veterans, Military exposures, Hypertension, Diabetes, High cholesterol, ASCVD

## Abstract

Many 1990–1991 Gulf War Veterans (GWVs) were exposed to toxicants and environmental hazards during deployment, including oil well fire smoke, chemical/biological agents, pyridostigmine bromide (PB) pills, and pesticides. Multiple constituents of smoke are associated with increased risk for atherosclerotic cardiovascular diseases (ASCVD), and other toxic exposures have been associated with autonomic and lipid dysfunction. We used data from the Gulf War Era Cohort and Biorepository Study of veterans deployed to Gulf War in 1990–1991 (*n* = 942). We evaluated the association of deployment exposures (no, yes (1–6 days), (7–30 days), (31 + days), and not sure) with clinical risk factors (hypertension, diabetes, high cholesterol) and ASCVD using multivariable logistic regression. We adjusted for all clinical risk factors in the models to test the association of military exposures and ASCVD. We found that 7–30 days exposure to oil well fire smoke (OR: 2.95, CI: 1.40, 6.19), PB pills (OR: 2.37, CI: 1.06, 5.32), and chemical/biological agents (OR: 3.60, CI: 1.04, 12.51) were associated with ASCVD. Exposure to chemical/biological agents for 7–30 days was also associated with hypertension (OR: 4.18, CI: 1.48, 11.86) and for 31 + days was associated with ASCVD (OR: 4.24, CI:1.20, 14.94). The associations between oil well fire smoke and chemical/biological agents with ASCVD remained significant in models adjusting for clinical risk factors. For GWVs, exposure to oil well fire smoke, chemical/biological agents, and PB pills were associated with ASCVD. These exposures may represent population-specific risk enhancers for ASCVD and may be considered in individualized clinical risk assessment.

**Supplementary Information:**

The online version contains supplementary material available at 10.1007/s12012-025-10013-7.

## Background

Approximately 30–44% of the nearly 700000 U.S. military personnel who served in the 1990–1991 Gulf War developed a complex of chronic, unexplained symptoms [1–3]. This condition is commonly known as Gulf War illness (GWI) and is characterized by persistent fatigue, musculoskeletal, gastrointestinal, respiratory, dermatological, and neuropsychological symptoms [1, 2]. These symptoms are not explained by standard medical or psychiatric diagnoses or medical tests. GWI has persisted more than 30 years for many veterans and continues to significantly impact their health and functional status [3].

In addition to usual stressors of wartime deployment, veterans who served in the 1990–91 Gulf War theater encountered numerous potentially hazardous exposures. The exposure concerns include chemical nerve agents, dense smoke from over 600 Kuwaiti oil wells that burned through most of 1991, use of pesticides, pyridostigmine bromide (PB) pills, and more [4, 5]. The most consistently identified GWI risk factors across all Gulf War Veteran (GWV) population studies include PB pills (given as a protective measure against nerve agents) and different types of pesticides, many of which function as acetylcholinesterase inhibitors and can exert toxic effects in the brain and periphery [4–6]. Chemical nerve agents (sarin/cyclosarin) have been implicated in the development of GWI symptoms in multiple studies but exposure assessment has been difficult [5, 6]. Long-term adverse effects of repeated, low-level exposure to these GW toxicants have been demonstrated through multiple animal studies which also provide key insights into mechanisms by which these toxicants could lead to the persistent symptoms of GWI, many years after exposure [7–9].

Animal studies have shown that exposures to the most likely toxic risk factors for GWI may also be associated with autonomic nervous system alterations, cardiomyopathy, and hypertension [10, 11]. These toxins also appear to lead to dysregulation of lipid metabolism resulting in metabolic disturbances, oxidative stress, and inflammation [12, 13], are also associated with GWI, and may contribute to increased risk of hypertension, diabetes, high cholesterol and atherosclerotic cardiovascular disease (ASCVD), including ischemic heart disease, ischemic cerebrovascular disease, and peripheral arterial disease [12, 14, 15].

In addition to neurotoxicant exposures during the Gulf War, U.S. service members encountered elevated levels of airborne hazards from the desert environment, sandstorms, and oil well fire smoke. There were 730 oil wells detonated with explosives; 656 wells burned for many months, while 74 wells gushed oil resulting in troops exposed to dense smoke, soot, and unburned oil in food, water, and clothing [16]. Particulate matter and chemicals in these airborne hazards have established causal links with cardiovascular diseases in population-based studies, affecting blood pressure control, lipid metabolism, vascular function, and atherogenesis [17–19].

Despite numerous reasons to suspect an increased risk of cardiovascular disease in GWVs, few studies have explored this directly [20–22]. Our current study addresses these gaps in the literature by examining associations of GW-related exposures with ASCVD and established clinical risk factors for ASCVD.

## Methods

We evaluated coded survey data from the VA Cooperative Studies Program (CSP) 585 “Gulf War Era Cohort and Biorepository.” Study data included information on GWVs’ sociodemographic and military characteristics, deployment exposures, and diagnosed medical conditions. The study was approved by the Institutional Review Board of Baylor College of Medicine and the Research and Development Committee of Michael E. DeBakey VA Medical Center, Houston, TX.

The CSP 585 cohort includes 942 GWVs who deployed to the Persian Gulf War between August 1990 and July 1991. Veterans completed the CSP 585 survey between 2014 and 2016. Details about the cohort and data collection have previously been reported [23, 24].

### Study Variables

Exposure variables- The CSP 585 survey elicited deployment exposure question responses about smoke from oil well fires, chemical/biological warfare agents, PB pills, pesticide cream or liquid on skin, uniform treated with pesticides, and insect baits/no-pest strips in living areas as yes, no, or not sure. Veterans who responded ‘yes’ to a given exposure were then asked to identify exposure duration (1–6 days, 7–30 days, 31 + days). To explore dose–response associations between military exposures and outcomes, we created a five-category variable for exposures. Categories included “no,” “not sure,” and “yes” responses (“yes and 1–6 days,” “yes and 7–30 days,” and “yes and 31 + days”) to be used as part of our main analysis. A sensitivity analysis treating exposures as “yes,” “no,” and “not sure” is reported in supplemental materials.

Health outcome variables- Physician-diagnosed medical conditions reported by veterans on the survey included hypertension, diabetes, and high cholesterol. We created a composite ASCVD variable based on veterans’ reports of one or more of the following diagnosed conditions: Heart attack, coronary heart disease, stroke, transient ischemic attack, and peripheral vascular disease.

Covariates- Survey questions on demographic and military characteristics included age at time of survey, sex, race and ethnicity and branch of service. For purposes of analyses, military branch of service included Army, Navy, Air Force, Marine Corps, and “Other” category that included Coast Guard, National Guard, Merchant Marines, National Oceanic and Atmospheric Administration, and Public Health Service. The survey question *“in your lifetime, have you smoked a total of at least 100 cigarettes, cigars or pipes”* was used to capture a history of ever smoking. The survey questions “*what is your height,” in both feet and inches and “what is your weight (pounds)”* were used to calculate body mass index (BMI) for each veteran. All non-responses and discrepant replies were set to missing.

### Statistical Analysis

Descriptive analysis investigated the distribution of demographic and military characteristics among veterans with and without each health outcome. Group differences were assessed using chi-square tests or the Fisher’s exact test for categorical variables and t-tests for continuous variables.

Separate multivariable logistic regression models were utilized to evaluate independent associations of each duration of exposure with each health outcome (hypertension, diabetes, high cholesterol, ASCVD), adjusted for age at survey, sex, race and ethnicity, smoking history, and BMI as part of the main analysis. We further report as supplemental Table 1, the association using the “yes,” “no,” and “not sure” question on military exposures and each outcome of interest.

We ran separate multivariable logistic regression models for each military exposure with significant association with ASCVD in the primary analysis, adjusting for the same covariates and hypertension, diabetes, and high cholesterol.

All analyses were performed using SAS version 9.4 (SAS Institute, Inc., Cary, NC) and Stata version 18 (StataCorp, College Station, TX).

## Results

Among 942 GWVs who completed the CSP 585 survey, 450 (48%) reported being diagnosed with hypertension, 158 (17%) with diabetes, 489 (52%) with high cholesterol, and 103 (11%) reported one or more ASCVD conditions.

Demographic, military, and health characteristics of veterans with each outcome of interest are presented in Table 1. All clinical risk factors and ASCVD were most prevalent in older veterans and in males (*p* < 0.05). Higher proportions of Black and Hispanic GWVs reported hypertension and diabetes compared to other groups (*p* < 0.05). Veterans with all health outcomes of interest also had significantly higher mean BMI (*p* < 0.05). GWVs identified as having ASCVD were more likely to report having ever smoked (*p* < 0.05).

**Table 1:** Baseline characteristics of veterans deployed to the 1990–1991 Gulf War by ASCVD and clinical risk factors

	Hypertension		Diabetes		High Cholesterol		ASCVD	
	No (*n* = 489)	Yes (*n* = 450)	No (*n* = 773)	Yes (*n* = 158)	No (*n* = 445)	Yes (*n* = 489)	No (*n* = 836)	Yes (*n* = 103)
	*n* (%)	*n* (%)	*n* (%)	*n* (%)	*n* (%)	*n* (%)	*n* (%)	*n* (%)
Age								
40–49 years	234 (48) ^*^	145 (32)	345 (45) ^*^	34 (22)	224 (50) ^*^	153 (31)	367 (44) ^*^	13 (13)
50–59 years	176 (36)	163 (36)	280 (36)	54 (34)	150 (34)	187 (38)	305 (36)	34 (33)
60 + years	79 (16)	142 (32)	148 (19)	70 (44)	71 (16)	149 (30)	164 (20)	56 (54)
Sex								
Male	352 (73) ^*^	373 (84)	582 (76) ^*^	139 (89)	325 (74) ^*^	396 (83)	638 (77)	87 (85)
Female	132 (27)	70 (16)	183 (24)	17 (11)	117 (26)	84 (18)	187 (23)	15 (15)
Race & Ethnicity								
White, not Hispanic	343 (72) ^*^	256 (60)	518 (69) ^*^	80 (54)	296 (68)	299 (64)	537 (66)	62 (65)
Black, not Hispanic	72 (15)	94 (22)	123 (16)	39 (26)	83 (19)	83 (18)	147 (18)	19 (20)
Hispanic (any race)	37 (8)	48 (11)	68 (9)	17 (11)	34 (8)	51 (11)	77 (10)	8 (8)
Other	27 (6)	28 (7)	42 (6)	13(9)	22 (5)	32 (7)	48 (6)	7 (7)
Branch of service								
Army	209 (43)	214 (48)	344 (45)	77 (49)	195 (44)	226 (46)	374 (45)	49 (48)
Navy	85 (17)	70 (16)	131 (17)	22 (14)	86 (19)	69 (14)	141 (17)	14 (14)
Air Force	49 (10)	46 (10)	81 (10)	14 (9)	42 (9)	51 (10)	91 (11)	4 (4)
Marine Corps	75 (15)	50 (11)	106 (14)	18 (11)	60 (13)	64 (13)	112 (13)	14 (14)
Other	71 (15)	70 (16)	111 (14)	27 (17)	62 (14)	79 (16)	118 (14)	22 (21)
Military Component								
Active duty only	280 (58)	265 (59)	443 (58)	97 (62)	255 (58)	287 (59)	486 (59)	58 (56)
Both active duty and reserves	120 (25)	118 (26)	194 (25)	44 (28)	108 (25)	130 (27)	205 (25)	33 (32)
Reserves only	85 (18)	64 (14)	130 (17)	16 (10)	77 (18)	70 (14)	138 (17)	12 (12)
Lifetime Smoking History								
No, < 100 cigarettes, cigars, or pipes	241 (50)	199 (45)	364 (48)	71 (46)	223 (51)	215 (45)	405 (50)*	34 (34)
Yes, > 100 cigarettes, cigars, or pipes	239 (50)	239 (55)	393 (52)	83 (54)	213 (49)	262 (55)	412 (50)	66 (66)
Body Mass Index, mean (SD)	28.49* (4.98)	30.92 (5.72)	29.18* (5.23)	31.88 (6.06)	28.79* (5.19)	30.41 (5.62)	29.52* (5.46)	30.81 (5.48)

(*) denotes *P* < 0.05. Missing values have been removed. Column percentage reported

As shown in Table 2, most GWVs reported exposure to smoke from oil well fires (*n* = 519 [61%] and use of PB pills (*n* = 408 [53%]). In bivariate analysis we found hypertension was significantly (*p* < 0.05) associated with chemical/biological agents, PB pills, and uniforms treated with pesticides.

**Table 2:** Exposure status and duration of exposure of veterans with ASCVD and clinical risk factors

	Exposure	Hypertension		Diabetes Mellitus		High Cholesterol		ASCVD	
Exposure/Duration	(*n*)	No (*n* = 489)	Yes (*n* = 450)	No (*n* = 773)	Yes (*n* = 158)	No (*n* = 445)	Yes (*n* = 489)	No (*n* = 836)	Yes (*n* = 103)
		*n* (%)	*n* (%)	*n* (%)	*n* (%)	*n* (%)	*n* (%)	*n* (%)	*n* (%)
Oil Well fires									
No	194	105 (24)	88 (21)	149 (21)	42 (29)	100 (25)	92 (21)	177 (24)	16 (17)
Yes									
1–6 days	113	63 (14)	49 (12)	90 (13)	23 (16)	56 (14)	56 (13)	99 (13)	13 (14)
7–30 days	196	90 (21)	106 (26)	166 (24)	27 (18)	85 (21)	110 (25)	167 (22)	29 (31)
31 + days	210	107 (25)	103 (25)	173 (25)	35 (24)	92 (23)	117 (26)	187 (25)	22 (23)
Not sure	136	71 (16)	65 (16)	116 (17)	19 (13)	65 (16)	71 (16)	122 (16)	14 (15)
Chemical/biological agents									
No	234	135 (30) ^*^	98 (24)	191 (27)	41 (28)	118 (28)	114 (25)	213 (27)	20 (23)
Yes									
1–6 days	51	19 (4)	32 (8)	40 (6)	10 (7)	23 (6)	27 (6)	44 (6)	7 (8)
7–30 days	26	7 (2)	19 (5)	19 (3)	7 (5)	9 (2)	17 (4)	21 (3)	5 (6)
31 + days	28	12 (3)	16 (4)	24 (3)	4 (3)	16 (4)	12 (3)	22 (3)	6 (7)
Not sure	531	282 (62)	248 (60)	444 (62)	82 (57)	250 (60)	279 (62)	480 (62)	50 (57)
Pyridostigmine bromide pills									
No	220	125 (31)^*^	94 (26)	186 (29)	31 (24)	109 (30)	109 (27)	202 (29)	17 (23)
Yes									
1–6 days	113	54 (13)	59 (16)	97 (15)	16 (13)	51 (14)	61 (15)	99 (14)	14 (19)
7–30 days	131	78 (19)	52 (14)	110 (17)	19 (15)	58 (16)	70 (17)	112 (16)	18 (24)
31 + days	164	85 (21)	79 (22)	134 (21)	29 (23)	84 (23)	80 (20)	155 (22)	9 (12)
Not sure	141	62 (15)	79 (22)	107 (17)	32 (25)	60 (17)	81 (20)	125 (18)	16 (22)
Pesticide cream on skin									
No	373	188 (43)	184 (46)	301 (44)	68 (47)	178 (44)	194 (45)	331 (44)	41 (48)
Yes									
1–6 days	37	21 (5)	16 (4)	29 (4)	7 (5)	21 (5)	16 (4)	32(4)	5 (6)
7–30 days	84	45 (10)	38 (10)	72 (10)	11(8)	44 (11)	38 (9)	77 (10)	6 (7)
31 + days	198	105 (24)	93 (23)	163 (24)	33 (23)	97 (24)	101 (23)	181 (24)	16 (19)
Not sure	148	80 (18)	68 (17)	122 (18)	25 (17)	62 (15)	85 (20)	130 (17)	18 (21)
Wore uniform treated with pesticides									
No	388	195 (43) ^*^	192 (46)	308 (43)	76 (51)	176 (43)	209 (46)	340 (44)	47 (51)
Yes									
1–6 days	8	4 (1)	4 (1)	7 (1)	1 (1)	7 (2)	1 (0.2)	8 (1)	0 (0)
7–30 days	23	11(2)	11(3)	21 (3)	2 (1)	14 (3)	8 (2)	20 (3)	2 (2)
31 + days	128	83 (18)	45 (11)	109 (15)	19 (13)	61 (15)	67 (15)	118 (15)	10 (11)
Not sure	323	160 (35)	163 (39)	269 (38)	50 (34)	156 (38)	166 (37)	290 (37)	33 (36)
Insect baits in living areas									
No	366	186 (42)	178 (44)	296 (42)	69 (49)	162 (40)	200 (46)	320 (42)	44 (50)
Yes									
1–6 days	6	3 (1)	3 (1)	3 (0.4)	3 (2)	3 (1)	3 (1)	5 (1)	1 (1)
7–30 days	21	11 (2)	10 (2)	18 (3)	3(2)	10 (2)	11 (3)	20 (3)	1 (1)
31 + days	235	129 (29)	106 (26)	198 (28)	34 (24)	121 (30)	113 (26)	216 (28)	19 (22)
Not sure	223	114 (26)	109 (27)	186 (27)	33 (23)	112 (27)	110 (25)	200 (26)	23 (26)

(*) denotes *P* < 0.05. Missing values have been removed. Column percentage reported

Using multivariable logistic regression models, we tested our a priori hypothesis that duration of exposure was associated with higher risk of health outcomes of interest controlling for confounding factors (Table 3). We could not test for associations between ASCVD and wore uniform treated with pesticides and any health outcomes and insect baits in living areas due to low sample size in the categories for duration of exposures. We found ASCVD was significantly associated with 7–30 days of exposure to smoke from oil well fires (OR 2.95; 95% CI: 1.40, 6.19), 7–30 days of exposure to PB pills (OR 2.37; 95% CI 1.06, 5.32), and both 7–30 days and 31 + days of exposure to chemical/biological warfare agents. Hypertension was also significantly associated with 7–30 days of exposure to chemical/biological agents (OR 4.18; 95% CI 1.48, 11.86). Our sensitivity analysis (supplemental Table 1) using exposures as “yes,” “no,” and “not sure” corroborated our significant findings of ASCVD and exposure to oil well fires (OR 2.05, 95% CI 1.06, 3.97) and hypertension and chemical/biological agents (OR 1.97, 95% CI 1.20, 3.22).

**Table 3:** Adjusted associations of ASCVD and clinical risk factors with military exposures based on exposure status and duration of exposure

	Hypertension	Diabetes	High Cholesterol	ASCVD
	aOR (95% CI)	aOR (95% CI)	aOR (95% CI)	aOR (95% CI)
Oil well fires				
No	1.0 (reference)	1.0 (reference)	1.0 (reference)	1.0 (reference)
Yes & 1–6 days	0.86 (0.51, 1.45)	0.94 (0.48, 1.82)	1.10 (0.67, 1.83)	2.02 (0.86, 4.77)
Yes & 7–30 days	1.48 (0.95, 2.31)	0.70 (0.39, 1.27)	1.55 (1.00, 2.40)	2.95 (1.40, 6.19)
Yes & 31 + days	1.09 (0.71, 1.70)	0.77 (0.43, 1.37)	1.49 (0.97, 2.28)	1.56 (0.71, 3.44)
Not sure	1.32 (0.80, 2.17)	0.74 (0.37, 1.47)	1.44 (0.89, 2.34)	2.08 (0.87, 4.93)
Chemical/biological agents				
No	1.0 (reference)	1.0 (reference)	1.0 (reference)	1.0 (reference)
Yes & 1–6 days	1.93 (0.95, 3.93)	1.19 (0.51, 2.82)	1.19 (0.59, 2.38)	1.21 (0.39, 3.67)
Yes & 7–30 days	4.18 (1.48, 11.86)	1.60 (0.54, 4.79)	2.19 (0.84, 5.69)	3.60 (1.04, 12.51)
Yes & 31 + days	1.96 (0.78, 4.93)	0.83 (0.23, 3.0)	0.97 (0.39, 2.39)	4.24 (1.20, 14.94)
Not sure	1.17 (0.83, 1.66)	0.93 (0.58, 1.49)	1.26 (0.90, 1.77)	1.29 (0.71, 2.37)
Pyridostigmine bromide pills				
No	1.0 (reference)	1.0 (reference)	1.0 (reference)	1.0 (reference)
Yes & 1–6 days	1.20 (0.72, 1.99)	0.89 (0.43, 1.85)	1.11 (0.67, 1.83)	1.32 (0.56, 3.13)
Yes & 7–30 days	0.80 (0.49, 1.31)	1.19 (0.59, 2.41)	1.18 (0.73, 1.90)	2.37 (1.06, 5.32)
Yes & 31 + days	0.96 (0.61, 1.53)	1.31 (0.69, 2.49)	0.84 (0.54, 1.33)	0.74 (0.29, 1.82)
Not sure	1.51 (0.94, 2.43)	1.86 (0.98, 3.52)	1.35 (0.85, 2.16)	1.59 (0.71, 3.58)
Pesticide cream on skin				
No	1.0 (reference)	1.0 (reference)	1.0 (reference)	1.0 (reference)
Yes & 1–6 days	0.72 (0.34, 1.52)	0.87 (0.34, 2.24)	0.65 (0.31, 1.36)	1.17 (0.41, 3.39)
Yes & 7–30 days	0.80 (0.47, 1.37)	0.76 (0.36, 1.62)	0.84 (0.49, 1.42)	0.81 (0.31, 2.11)
Yes & 31 + days	0.95 (0.64, 1.40)	1.05 (0.62, 1.79)	1.07 (0.73, 1.57)	0.87 (0.44, 1.74)
Not sure	0.77 (0.50, 1.19)	0.98 (0.55, 1.75)	1.44 (0.94, 2.19)	1.58 (0.82, 3.04)
Wore uniform treated with pesticides				
No	1.0 (reference)	1.0 (reference)	1.0 (reference)	–
Yes & 1–6 days	1.00 (0.23, 4.42)	0.54 (0.06, 5.07)	0.10 (0.01, 0.89)	–
Yes & 7–30 days	1.40 (0.51, 3.81)	0.54 (0.07, 4.30)	0.73 (0.26, 1.99)	–
Yes & 31 + days	0.51 (0.32, 0.82)	0.83 (0.44, 1.54)	1.02 (0.66, 1.59)	–
Not sure	1.09 (0.79, 1.53)	0.79 (0.50, 1.24)	0.98 (0.71, 1.36)	–
Insect baits in living areas				
No	–	–	–	–
Yes & 1–6 days	–	–	–	–
Yes & 7–30 days	–	–	–	–
Yes & 31 + days	–	–	–	–
Not sure	–	–	–	–

Adjusted for age, sex, race and ethnicity, body mass index, and smoking. Missing values have been removed for analysis. Bold text denotes statistical significance. *ASCVD* Atherosclerotic Cardiovascular Disease, *aOR* Adjusted Odds Ratio. Due to small cell size numbers adjusted analysis using exposure to uniform treated with pesticides and insect baits in living areas could not be performed

Since we found significant associations of ASCVD with oil well fires, chemical/biological agents, and PB pills in our main analysis, we further tested for associations between ASCVD and each duration of exposure in separate multivariable logistic regression models adjusting for the clinical risk factors of hypertension, high cholesterol, and diabetes in addition to other covariates (Table 4). We found that the associations between ASCVD and 7–30 days of exposure to smoke from oil well fires (OR 2.32; 95% CI 1.07, 5.03) and 31 + days of exposure to chemical/biological agents (OR 4.49; 95% CI 1.18, 17.18) remained statistically significant. In all these models, high cholesterol was significantly associated (*P* < 0.05) indicating a possible mediating role in the association between ASCVD and smoke from oil well fires and chemical/biological agents (Supplemental Table 2).

**Table 4:** Adjusted associations of ASCVD with military exposures based on exposure status and duration of exposure adjusting for all clinical risk factors

	ASCVD
	aOR (95% CI)
Oil well fires	
No	1.0 (reference)
Yes & 1–6 days	1.95 (0.79, 4.76)
Yes & 7–30 days	2.32 (1.07, 5.03)
Yes & 31 + days	1.41 (0.62, 3.18)
Not sure	1.79 (0.73, 4.36)
Chemical/biological agents	
No	1.0 (reference)
Yes & 1–6 days	1.16 (0.37, 3.63)
Yes & 7–30 days	2.65 (0.76, 9.29)
Yes & 31 + days	4.49 (1.18, 17.18)
Not sure	1.11 (0.59, 2.07)
Pyridostigmine bromide pills	
No	1.0 (reference)
Yes & 1–6 days	1.37 (0.57, 3.31)
Yes & 7–30 days	2.23 (0.95, 5.25)
Yes & 31 + days	0.73 (0.29, 1.84)
Not sure	1.39 (0.61, 3.18)

Adjusted for age, sex, race and ethnicity, body mass index, smoking, hypertension, high cholesterol, and diabetes. Bold text denotes statistical significance. *ASCVD* Atherosclerotic Cardiovascular Disease, *aOR* Adjusted Odds Ratio

## Discussion

In this national cohort of GWVs (*n* = 942), we found significant associations between ASCVD and veteran-reported exposure to smoke from oil well fires, chemical/biological agents and PB pills and between hypertension and exposure to chemical/biological agents. Preclinical research supports the biological plausibility of these associations, although more research is needed to substantiate our findings that GW exposures may be considered ASCVD risk enhancers. Our findings were bolstered by additional analyses in which we found significant association between extended exposure to smoke from oil well fires and chemical/biological agents with ASCVD after controlling for the established clinical risk factors of hypertension, high cholesterol, and diabetes. Our results are largely consistent with the limited published literature and current understanding of pathophysiological effects of the evaluated exposures on cardiometabolic health.

Our study is important in this aging, largely male cohort. Deployed veterans’ mean age at time of completion of the CSP 585 survey was 53 years. Age is an established risk factor for developing ASCVD in the general population [25, 26]. There is an earlier onset and a higher risk of coronary artery disease among males until the onset of menopause in females [27]. Exposure to cholinesterase-inhibiting chemicals like PB pills and organophosphate and carbamate pesticides are suspected causal contributors to GWI and have been shown to elicit autonomic imbalance, metabolic disturbances, and lipid dysfunction in animal models [10, 12, 13]. Chemicals such as arsenic, lead, cadmium, volatile organic compounds, and particulate matter found in smoke from oil well fires can increase risk of cardiovascular morbidity and mortality [17–19].

Our findings support the limited literature indicating that military exposures encountered in the Gulf War may elevate the risk of ASCVD [20]. The 2013–2014 survey of GWVs in the Fort Devens Cohort (*n* = 448) explored chronic disease outcomes in this sample of GWVs. Investigators found GWVs who reported exposure to chemical/biological agents, had 1.60 higher odds (95% CI: 1.01, 2.53) of developing hypertension, and 3.24 higher odds (95% CI: 1.65, 6.38) of developing diabetes compared to unexposed GWVs [20]. They further found significant associations between PB pills and heart attack and diabetes. In our analysis, we found significant association between longer duration PB pills and ASCVD and exposure to chemical/biological agents and hypertension. However, in our cohort, we did not identify associations between diabetes and any of the exposures of interest.

The Department of Veteran Affairs (VA) provides healthcare and can award disability benefits to veterans based on a presumed connection between military service and an adverse health outcome (a “presumptive condition”). The findings from this study indicate that ASCVD may be a candidate for presumed service connection in GWVs. In fact, the VA has recognized ischemic heart disease (IHD) as a presumptive condition for veterans who served in the Vietnam War between 1962 and 1975 based on research linking dioxin in Agent Orange to IHD [28, 29]. Similar to GWVs and their exposures, Vietnam-era veterans were unable to document actual exposure to Agent Orange. Designating diagnosed diseases, such as cardiovascular disease, to be presumptive conditions by policy or law removes the barrier of documenting actual exposures for veterans seeking care and support from VA. No such presumed service connection exists for ASCVD for GWVs currently, although evidence supporting an association between ASCVD, and one or more GW deployment-related exposure is growing [20, 22].

Clinicians may also want to be vigilant for symptoms of undiagnosed ASCVD and implement standard preventive interventions and consider earlier screenings with GWVs exposed to smoke from oil well fires, chemical/biological agents and PB pills. This would require obtaining a history of military and civilian exposures of relevance and using more aggressive screening measures based on presence of exposures possibly related to ASCVD, treating these exposures as “ASCVD risk enhancers.” Increasing age and the predominantly male sex point to an elevated baseline risk of ASCVD in the GWV cohort [22, 25, 26, 30]. As the evidence of causal association accumulates, policies and clinical standards may need to shift to mitigate the increased risk of ASCVD related to military exposures.

Our study has important strengths. The CSP 585 survey had clear and concise questions on military exposures, sociodemographics, and military service characteristics, as well as our outcomes of interest. The cohort is relatively large and includes veterans from across the US. This study also has some limitations which merit consideration. There may be difficulty remembering the military exposure history since the survey was completed nearly 25 years after the Gulf War. Further, there may be selection bias since veterans with high disease burden may have been more motivated to participate in the CSP 585 survey compared to healthier veterans. Despite the large sample overall, the infrequency of some outcomes limited certain statistical testing, i.e., the association between uniform treated with pesticides and ASCVD and insect baits in living areas and any of the health outcomes could not be tested. These limitations may also explain the apparent inconsistencies of some findings, such as why only intermediate duration of exposure to smoke from oil well fires and PB pills were significantly associated with some health outcomes. The CSP 585 survey did not have a specific question on physician-diagnosed peripheral arterial disease, and we instead used “peripheral vascular disease” to make the composite ASCVD variable. The CSP 585 survey did not have specific question on tobacco use based on pack years therefore we used the survey question “In your lifetime, have you smoked a total of at least 100 cigarettes, cigars or pipes?” We acknowledge that while the models adjust for known risk factors, there may be unmeasured confounders which may influence the point estimate of the association in unpredictable ways. The unexplained variability likely contributes to the wide confidence intervals seen in some comparisons, making it more likely that our analyses miss a true association, i.e., biases our findings toward a null finding (false negative). These limitations notwithstanding, this focused analysis of the association of GW exposures with ASCVD and its clinical risk factors provides important new insights, and replication in larger samples is warranted.

In conclusion, our findings demonstrate that GWVs with exposure to smoke from oil well fires, chemical/biological agents, and PB pills have higher odds of reporting ASCVD which may merit consideration as a presumptive service-connected condition. Clinicians should explore these exposures as possible risk enhancers in shared decision-making conversations with their Gulf War veteran patients to consider more proactive ASCVD screening and preventive measures.

## Supplementary Information

Below is the link to the electronic supplementary material.

Supplementary file1 (DOCX 21 KB)

## Data Availability

Data cannot be made available to other researchers because of the nature of the data and Veteran Affairs privacy rules.

## References

[1] Steele, L. (2000). Prevalence and patterns of Gulf War illness in Kansas veterans: Association of symptoms with characteristics of person, place, and time of military service. *American Journal of Epidemiology,**152*(10), 992–1002. 10.1093/aje/152.10.992 11092441 10.1093/aje/152.10.992

[2] Fukuda, K., Nisenbaum, R., Stewart, G., Thompson, W. W., Robin, L., Washko, R. M., Noah, D. L., Barrett, D. H., Randall, B., Herwaldt, B. L., Mawle, A. C., & Reeves, W. C. (1998). Chronic multisymptom illness affecting air force veterans of the Gulf War. *JAMA,**280*(11), 981–988. 10.1001/jama.280.11.981 9749480 10.1001/jama.280.11.981

[3] Ahmed, S. T., Steele, L., Richardson, P., Nadkarni, S., Bandi, S., Rowneki, M., Sims, K. L., Vahey, J., Gifford, E. J., Boyle, S. H., Nguyen, T. H., Djosta, A. N., White, D. L., Hauser, E. R., Chandler, H., Yamal, J. M., & Helmer, D. A. (2022). Association of Gulf War illness-related symptoms with military exposures among 1990–1991 Gulf War veterans evaluated at the war-related illness and injury study center (WRIISC). *Brain Sciences,**12*(3), 321. 10.3390/brainsci12030321 35326276 10.3390/brainsci12030321 PMC8946608

[4] Research Advisory Committee Report on Gulf War Illnesses. *Gulf War Illness and the Health of Gulf War Veterans: Research Update and Recommendations, 2009–2013*. U.S. Government Printing Office Washington, D.C. 2014. Accessed April 2022

[5] Institute of Medicine (US) Committee on Gulf War and Health: Health Effects of Serving in the Gulf War, Update 2009. “Gulf War and Heath: Volume 8: Update of Health Effects of Serving in the Gulf War”. Washington (DC): National Academics Press (US); 2010 Appendix A, Cholinesterase Inhibitors and Multi symptom Illness. Accessed May 2022

[6] Proctor, S. P., Heeren, T., White, R. F., Wolfe, J., Borgos, M. S., Davis, J. D., Pepper, L., Clapp, R., Sutker, P. B., Vasterling, J. J., & Ozonoff, D. (1998). Health status of Persian Gulf War veterans: Self-reported symptoms, environmental exposures and the effect of stress. *International Journal of Epidemiology,**27*(6), 1000–1010. 10.1093/ije/27.6.1000 10024195 10.1093/ije/27.6.1000

[7] Abdullah, L., Evans, J. E., Joshi, U., Crynen, G., Reed, J., Mouzon, B., Baumann, S., Montague, H., Zakirova, Z., Emmerich, T., Bachmeier, C., Klimas, N., Sullivan, K., Mullan, M., Ait-Ghezala, G., & Crawford, F. (2016). Translational potential of long-term decreases in mitochondrial lipids in a mouse model of Gulf War Illness. *Toxicology,**372*, 22–33. 10.1016/j.tox.2016.10.012 27931520 10.1016/j.tox.2016.10.012

[8] Ribeiro, A. C. R., & Deshpande, L. S. (2021). A review of pre-clinical models for Gulf War illness. *Pharmacology and Therapeutics,**228*, 107936. 10.1016/j.pharmthera.2021.107936 34171340 10.1016/j.pharmthera.2021.107936

[9] Mozhui, K., O’Callaghan, J. P., Ashbrook, D. G., Prins, P., Zhao, W., Lu, L., & Jones, B. C. (2023). Epigenetic analysis in a murine genetic model of Gulf War illness. *Frontiers in Toxicology,**5*, 1162749. 10.3389/ftox.2023.1162749 37389175 10.3389/ftox.2023.1162749 PMC10300436

[10] Bernátová, I., Babál, P., Grubbs, R. D., & Morris, M. (2006). Acetylcholinesterase inhibition affects cardiovascular structure in mice. *Physiological Research,**55*(Suppl 1), S89–S97. 10.33549/physiolres.930000.55.S1.89 17177630 10.33549/physiolres.930000.55.S1.89

[11] Shewale, S. V., Anstadt, M. P., Horenziak, M., Izu, B., Morgan, E. E., Lucot, J. B., & Morris, M. (2012). Sarin causes autonomic imbalance and cardiomyopathy: An important issue for military and civilian health. *Journal of Cardiovascular Pharmacology,**60*(1), 76–87. 10.1097/FJC.0b013e3182580b75 22549449 10.1097/FJC.0b013e3182580b75

[12] Emmerich, T., Zakirova, Z., Klimas, N., Sullivan, K., Shetty, A. K., Evans, J. E., Ait-Ghezala, G., Laco, G. S., Hattiangady, B., Shetty, G. A., Mullan, M., Crynen, G., Abdullah, L., & Crawford, F. (2017). Phospholipid profiling of plasma from GW veterans and rodent models to identify potential biomarkers of Gulf War Illness. *PLoS ONE,**12*(4), e0176634. 10.1371/journal.pone.0176634 28453542 10.1371/journal.pone.0176634 PMC5409146

[13] Jean-Pierre, M., Michalovicz, L. T., Kelly, K. A., O’Callaghan, J. P., Nathanson, L., Klimas, N., & Craddock, T. J. (2022). A pilot reverse virtual screening study suggests toxic exposures caused long-term epigenetic changes in Gulf War illness. *Computational and Structural Biotechnology Journal,**20*, 6206–6213. 10.1016/j.csbj.2022.11.006 36420170 10.1016/j.csbj.2022.11.006 PMC9676197

[14] Naviaux, R. K., Naviaux, J. C., Li, K., Wang, L., Monk, J. M., Bright, A. T., Koslik, H. J., Ritchie, J. B., & Golomb, B. A. (2019). Metabolic features of Gulf War illness. *PLoS ONE,**14*(7), e0219531. 10.1371/journal.pone.0219531 31348786 10.1371/journal.pone.0219531 PMC6660083

[15] Choi, R. H., Tatum, S. M., Symons, J. D., Summers, S. A., & Holland, W. L. (2021). Ceramides and other sphingolipids as drivers of cardiovascular disease. *Nature Reviews Cardiology,**18*(10), 701–711. 10.1038/s41569-021-00536-1 33772258 10.1038/s41569-021-00536-1 PMC8978615

[16] *The CDC Conference on the Health Impacts of Chemical Exposures during the Gulf War - OIL FIRES, PETROLEUM and GULF WAR ILLNESS*. (1999). Accessed October 28, 2024, from https://ngwrc.org/docs/Study/CDC-1999-Confrence-Oilwell-Fires-&-GWI.pdf

[17] Cosselman, K. E., Navas-Acien, A., & Kaufman, J. D. (2015). Environmental factors in cardiovascular disease. *Nature Reviews Cardiology,**12*(11), 627–642. 10.1038/nrcardio.2015.152 26461967 10.1038/nrcardio.2015.152

[18] Lamas, G. A., Bhatnagar, A., Jones, M. R., Mann, K. K., Nasir, K., Tellez-Plaza, M., Ujueta, F., & Navas-Acien, A. (2023). Contaminant metals as cardiovascular risk factors: A Scientific statement from the American heart association. *Journal of the American Heart Association,**12*(13), e029852. 10.1161/JAHA.123.029852 37306302 10.1161/JAHA.123.029852 PMC10356104

[19] Rajagopalan, S., & Landrigan, P. J. (2021). Pollution and the heart. *New England Journal of Medicine,**385*(20), 1881–1892. 10.1056/NEJMra2030281 34758254 10.1056/NEJMra2030281

[20] Zundel, C. G., Krengel, M. H., Heeren, T., Yee, M. K., Grasso, C. M., Lloyd, P. A. J., Coughlin, S. S., & Sullivan, K. (2019). Rates of chronic medical conditions in 1991 Gulf War veterans compared to the general population. *International Journal of Environmental Research and Public Health,**16*(6), 949. 10.3390/ijerph16060949 30884809 10.3390/ijerph16060949 PMC6466358

[21] Zundel, C. G., Heeren, T., Grasso, C. M., Spiro, A., III., Proctor, S. P., Sullivan, K., & Krengel, M. (2020). Changes in health status in the Ft. Devens gulf war veterans cohort: 1997–2017. *Neuroscience Insights,**15*, 2633105520952675. 10.1177/2633105520952675 32914090 10.1177/2633105520952675 PMC7444112

[22] Ahmed, S. T., Li, R., Richardson, P., Ghosh, S., Steele, L., White, D. L., Djotsa, A. N., Sims, K., Gifford, E., Hauser, E. R., Virani, S. S., Morgan, R., Delclos, G., & Helmer, D. A. (2023). Association of atherosclerotic cardiovascular disease, hypertension, diabetes, and hyperlipidemia with gulf war illness among gulf war veterans. *Journal of the American Heart Association,**12*(19), e029575. 10.1161/JAHA.123.029575 37772504 10.1161/JAHA.123.029575 PMC10727258

[23] Khalil, L., McNeil, R. B., Sims, K. J., Felder, K. A., Hauser, E. R., Goldstein, K. M., Voils, C. I., Klimas, N. G., Brophy, M. T., Thomas, C. M., Whitley, R. L., Dursa, E. K., Helmer, D. A., & Provenzale, D. T. (2018). The Gulf War Era cohort and biorepository: A longitudinal research resource of veterans of the 1990–1991 Gulf War Era. *American Journal of Epidemiology,**187*(11), 2279–2291. 10.1093/aje/kwy147 30060060 10.1093/aje/kwy147

[24] Gifford, E. J., Vahey, J., Hauser, E. R., Sims, K. J., Efird, J. T., Dursa, E. K., Steele, L., Helmer, D. A., & Provenzale, D. (2021). Gulf War illness in the gulf war era cohort and biorepository: The Kansas and Centers for disease control definitions. *Life Sciences,**278*, 119454. 10.1016/j.lfs.2021.119454 33811897 10.1016/j.lfs.2021.119454 PMC10148241

[25] “Center for Disease Control and Prevention. Know your risk for heart disease”. https://www.cdc.gov/heartdisease/risk_factors.htm. Accessed May 2022

[26] Rodgers, J. L., Jones, J., Bolleddu, S. I., Vanthenapalli, S., Rodgers, L. E., Shah, K., Karia, K., & Panguluri, S. K. (2019). Cardiovascular risks associated with gender and aging. *Journal of Cardiovascular Development and Disease,**6*(2), 19. 10.3390/jcdd6020019 31035613 10.3390/jcdd6020019 PMC6616540

[27] Iorga, A., Cunningham, C. M., Moazeni, S., Ruffenach, G., Umar, S., & Eghbali, M. (2017). The protective role of estrogen and estrogen receptors in cardiovascular disease and the controversial use of estrogen therapy. *Biology of Sex Differences,**8*(1), 33. 10.1186/s13293-017-0152-8 29065927 10.1186/s13293-017-0152-8 PMC5655818

[28] U.S. Department of Veterans Affairs. (2020). “Agent orange presumptive conditions”. Retrieved May, 2022, from https://www.publichealth.va.gov/exposures/publications/agent-orange/agent-orange-2020/presumptive.asp

[29] U.S. Department of Veterans Affairs. (2022). “Presumptive disability benefits”. Retrieved October, 2022, from https://www.benefits.va.gov/BENEFITS/factsheets/serviceconnected/presumption.pdf

[30] Duong, L. M., Nono Djotsa, A. B., Vahey, J., Steele, L., Quaden, R., Harrington, K. M., Ahmed, S. T., Polimanti, R., Streja, E., Gaziano, J. M., & Concato, J. (2022). Association of Gulf war illness with characteristics in deployed vs. non-deployed Gulf war era veterans in the cooperative studies program 2006/million veteran program 029 cohort: A cross-sectional analysis. *International Journal of Environmental Research and Public Health,**20*(1), 258. 36612580 10.3390/ijerph20010258 PMC9819371

