# Supplementary material for: Atherosclerotic Cardiovascular Disease in Gulf War Veterans in Relation to Deployment Exposures
Source: Cardiovasc Toxicol. 2025 Jul 5;25(9):1263–71. doi: 10.1007/s12012-025-10013-7 (PMC12310855; doi:10.1007/s12012-025-10013-7)
Supplement: Supplementary file 1 — Supplementary file1 (DOCX 21 KB) [file 12012_2025_10013_MOESM1_ESM.docx]

Supplemental Table 1: Adjusted associations of ASCVD and clinical risk factors with military exposures of interest

|  | **Exposure status** | **Hypertension** | **Diabetes** | **High**  **Cholesterol** | **ASCVD** |
| --- | --- | --- | --- | --- | --- |
|  | n | aOR (95% CI) | aOR (95% CI) | aOR (95% CI) | aOR (95% CI) |
| **Oil well fires** |  |  |  |  |  |
| No | 194 | 1.0 (reference) | 1.0 (reference) | 1.0 (reference) | 1.0 (reference) |
| Yes | 562 | 1.13 (0.78, 1.64) | 0.78 (0.49, 1.25) | 1.38 (0.96, 1.98) | 2.05 (1.06, 3.97) |
| Not sure | 136 | 1.35 (0.82, 2.22) | 0.73 (0.37, 1.45) | 1.45 (0.89, 2.36) | 2.15 (0.90, 5.10) |
| **Chemical/biological agents** |  |  |  |  |  |
| No | 234 | 1.0 (reference) | 1.0 (reference) | 1.0 (reference) | 1.0 (reference) |
| Yes | 137 | 1.97 (1.20, 3.22) | 1.18 (0.63, 2.19) | 1.33 (0.82, 2.15) | 2.10 (1.00, 4.43) |
| Not sure | 531 | 1.18 (0.83, 1.67) | 0.94 (0.58, 1.49) | 1.25 (0.89, 1.75) | 1.26 (0.69, 2.31) |
| **Pyridostigmine bromide pills** |  |  |  |  |  |
| No | 220 | 1.0 (reference) | 1.0 (reference) | 1.0 (reference) | 1.0 (reference) |
| Yes | 534 | 1.01 (0.71, 1.44) | 1.10 (0.66, 1.85) | 1.06 (0.75, 1.50) | 1.56 (0.83, 2.91) |
| Not sure | 141 | 1.53 (0.95, 2.46) | 1.88 (1.00, 3.53) | 1.35 (0.84, 2.16) | 1.56 (0.70, 3.48) |
| **Wore uniform treated with pesticides** |  |  |  |  |  |
| No | 388 | 1.0 (reference) | 1.0 (reference) | 1.0 (reference) | 1.0 (reference) |
| Yes | 189 | 0.63 (0.42, 0.94) | 0.77 (0.44, 1.35) | 0.91 (0.62, 1.35) | 0.76 (0.38, 1.52) |
| Not sure | 323 | 1.09 (0.78, 1.52) | 0.78 (0.49, 1.22) | 0.98 (0.71, 1.36) | 1.01 (0.59, 1.73) |
| **Insect baits/no pest strips in living area** |  |  |  |  |  |
| No | 366 | 1.0 (reference) | 1.0 (reference) | 1.0 (reference) | 1.0 (reference) |
| Yes | 307 | 0.85 (0.60, 1.19) | 0.85 (0.54, 1.35) | 0.93 (0.67, 1.30) | 0.71 (0.40, 1.28) |
| Not sure | 223 | 0.97 (0.67, 1.41) | 0.82 (0.49, 1.39) | 0.85 (0.59, 1.23) | 1.14 (0.63, 2.09) |
| **Used pesticide cream/liquid on skin** |  |  |  |  |  |
| No | 373 | 1.0 (reference) | 1.0 (reference) | 1.0 (reference) | 1.0 (reference) |
| Yes | 379 | 0.89 (0.64, 1.24) | 0.87 (0.56, 1.35) | 0.97 (0.70, 1.33) | 1.01 (0.59, 1.74) |
| Not sure | 148 | 0.77 (0.50, 1.19) | 0.98 (0.55, 1.74) | 1.43 (0.94, 2.18) | 1.62 (0.84, 3.14) |

aOR: adjusted odds ratio. Logistic regression models adjusted for age at survey, sex, race & ethnicity, smoking and BMI.

Supplemental Table 2: Full models of ASCVD with military exposures based on exposure status and duration of exposure adjusting for all clinical risk factors

|  | **aOR (95% CI)** | **aOR (95% CI)** | **aOR (95% CI)** |
| --- | --- | --- | --- |
| **Exposure of interest per model** | **Oil well fire smoke** | **Chemical/biological agents** | **PB pills** |
| **No** | 1.0 (Reference) | 1.0 (Reference) | 1.0 (Reference) |
| **Yes & 1-6 days** | 1.95 (0.79, 4.76) | 1.16 (0.37, 3.63) | 1.37 (0.57, 3.31) |
| **Yes & 7-30 days** | 2.32 (1.07, 5.03) | 2.65 (0.76, 9.29) | 2.23 (0.95, 5.25) |
| **Yes & 31+ days** | 1.41 (0.62, 3.18) | 4.49 (1.18, 17.18) | 0.73 (0.29, 1.84) |
| **Not sure** | 1.79 (0.73, 4.36) | 1.11 (0.59, 2.07) | 1.39 (0.61, 3.18) |
| **Age** |  |  |  |
| **40-49 years** | 1.0 (Reference) | 1.0 (Reference) | 1.0 (Reference) |
| **50-59 years** | 2.75 (1.27, 5.93) | 3.59 (1.57, 8.27) | 4.94 (1.81, 13.48) |
| **60+ years** | 7.83 (3.59, 17.08) | 9.26 (3.99, 21.46) | 12.23 (4.39, 34.08) |
| **Sex** |  |  |  |
| **Male** | 1.0 (Reference) | 1.0 (Reference) | 1.0 (Reference) |
| **Female** | 0.78 (0.37, 1.65) | 0.56 (0.26, 1.21) | 0.69 (0.30, 1.60) |
| **Race & Ethnicity** |  |  |  |
| **White, not Hispanic** | 1.0 (Reference) | 1.0 (Reference) | 1.0 (Reference) |
| **Black, not Hispanic** | 1.07 (0.53, 2.16) | 0.84 (0.41, 1.72) | 0.89 (0.41, 1.95) |
| **Hispanic (any race)** | 1.15 (0.49, 2.70) | 1.23 (0.50, 3.02) | 0.97 (0.36, 2.58) |
| **Other** | 1.08 (0.42, 2.77) | 0.89 (0.31, 2.53) | 1.20 (0.44, 3.31) |
| **Lifetime Smoking History** |  |  |  |
| **No,** <**100 cigarettes, cigars, or pipes** | 1.0 (Reference) | 1.0 (Reference) | 1.0 (Reference) |
| **Yes, >100 cigarettes, cigars, or pipes** | 1.62 (0.96, 2.73) | 1.61 (0.94, 2.75) | 1.33 (0.75, 2.38) |
| **Body Mass Index, mean (SD)** | 1.04 (0.99, 1.09) | 1.03 (0.98, 1.08) | 1.03 (0.98, 1.09) |
| **Hypertension** | 1.49 (0.84, 2.68) | 1.70 (0.94, 3.08) | 1.72 (0.90, 3.27) |
| **Diabetes Mellitus** | 1.19 (0.65, 2.16) | 1.12 (0.61, 2.04) | 1.23 (0.64, 2.35) |
| **High Cholesterol** | 4.29 (2.16, 8.56) | 4.05 (2.02, 8.13) | 3.78 (1.76, 8.11) |

Adjusted for age, sex, race and ethnicity, body mass index, smoking, hypertension, high cholesterol, and diabetes. Bold text denotes statistical significance. ASCVD= atherosclerotic cardiovascular disease; aOR= adjusted odds ratio. Uniform treated with pesticides could not be tested and is not included in this table.
